# PROMISE: effect of protein supplementation on fat-free mass preservation after bariatric surgery, a randomized double-blind placebo-controlled trial

**DOI:** 10.1186/s13063-023-07654-w

**Published:** 2023-11-09

**Authors:** A. E. Taselaar, A. J. Boes, R. W. F. de Bruin, T. M. Kuijper, K. Van Lancker, E. van der Harst, R. A. Klaassen

**Affiliations:** 1 Maasstad Ziekenhuis Rotterdam, Maasstadweg 21, Rotterdam, 3079 DZ The Netherlands; 2 Department of Surgery, Erasmus MC, Erasmus MC Transplant Institute, University Medical Center, Rotterdam, Netherlands; 3 Maasstad Academy, Maasstad Hospital, Rotterdam, Netherlands; 4 Department of Applied Mathematics, Computer Science and Statistics, Ghent University, Ghent, Belgium

## Abstract

**Introduction:**

Protein malnutrition after bariatric surgery is a severe complication and leads to significant morbidity. Previous studies have shown that protein intake and physical activity are the most important factors in the preservation of fat-free mass during weight loss. Low protein intake is very common in patients undergoing bariatric surgery despite dietary counseling. Protein powder supplements might help patients to achieve the protein intake recommendations after bariatric surgery and could therefore contribute to preserve fat-free mass. This double-blind randomized placebo-controlled intervention study aims to assess the effect of a daily consumed clear protein powder shake during the first 6 months after bariatric surgery on fat-free mass loss in the first 12 months after laparoscopic Roux-en-Y gastric bypass (LRYGB).

**Methods and analysis:**

Inclusion will take place at the outpatient clinic of the bariatric expertise center for obesity of the Maasstad Hospital. Patients will be randomly assigned to either the intervention or control group before surgery. The intervention group will receive a clear protein powder shake of 200 ml containing 20 g of whey protein dissolved in water which should be taken daily during the first 6 months after LRYGB on top of their normal postoperative diet. The control group will receive an isocaloric, clear, placebo shake containing maltodextrine. Postoperative rehabilitation and physiotherapeutical guidance will be standardized and similar in both groups. Also, both groups will receive the same dietary advice from specialized dieticians. The main study parameter is the percentage of fat-free mass loss 6 months after surgery, assessed by multi-frequency bioelectrical impedance analysis (MF-BIA).

**Ethics and dissemination:**

The protocol, version 2 (February 20, 2022) has been approved by the Medical Research Ethics Committees United (MEC-U) (NL 80414.100.22). The results of this study will be submitted to peer-reviewed journals.

**Trial registration:**

ClinicalTrials.gov NCT05570474. Registered on October 5, 2022.

**Supplementary Information:**

The online version contains supplementary material available at 10.1186/s13063-023-07654-w.

## Highlights

• First RCT to compare clear protein shakes to placebo after bariatric surgery

• Study conducted in a specialized center performing large volumes of bariatric procedures

• This RCT may improve the postoperative treatment of bariatric patients significantly

**Supplementary Information:**

The online version contains supplementary material available at 10.1186/s13063-023-07654-w.

## Background

### Bariatric surgery

Bariatric surgery is considered the most effective treatment for morbid obesity. It results in significant weight loss and reduces obesity-related comorbidities. Unfortunately, bariatric surgery can also result in long-term complications such as vitamin- and mineral deficiencies, protein malnutrition, and excessive loss of fat-free mass [1, 2]. These complications are due to a decreased intake and altered absorption and digestion after surgery, as a result of the decreased gastric capacity and rapid passage through the gastro-intestinal tract.

### Fat-free mass preservation

Protein malnutrition is a severe complication and leads to increased morbidity [1]. Previous studies have shown that protein intake and physical activity are the most important factors in the preservation of fat-free mass during weight loss [3]. Low protein intake after bariatric surgery is very common [4] due to the aversion of protein-rich foods, a decrease in overall food intake, and food intolerances. As previous studies have shown, low protein intake can lead to excessive loss of fat-free mass, which is seen in 14–46% of bariatric patients [5]. Since fat-free mass consists of 30–50% muscle mass, it is crucial for several metabolic mechanisms like resting energy expenditure, preservation of bone strength, and recovery from stressful situations [6]. These metabolic mechanisms can contribute to the desired long-term results of bariatric surgery such as persistent weight loss, improved quality of life, and reduction of comorbidities. Excessive loss of fat-free mass therefore decreases the beneficial effect of bariatric surgery and should be prevented.

### Protein supplementation

Thus, a protein-enriched diet is advised by specialized dietitians. Despite these recommendations, less than half of the patients at our institute achieve the recommendation to consume at least 60 g of protein per day. As the pilot study of Schollenberger et al. shows, a protein powder supplement can be used to increase protein intake after bariatric surgery without a negative impact on renal function [7]. The results of this pilot study suggest that protein powder supplementation leads to increased body fat loss and fat-free mass preservation after bariatric surgery. However, due to a small sample size, the results did not reach statistical significance. In addition, it has previously been shown that protein supplements might be absorbed better than dietary proteins [8].

As previous research indicates that most of the fat-free mass loss occurs in the first 3 to 6 months after surgery [5], it seems important to investigate whether protein powder supplementation affects fat-free mass loss in the early postoperative period. From clinical practice, we know that patients often experience difficulties with taking protein shakes, because they have to be dissolved in milk. Milk and dairy-like products are often not tolerated well postoperatively, due to changes in food preferences, taste, and smell following bariatric surgery [9].

### Study

In light of these considerations, the aim of this study is to assess the effect of a daily consumed shake of protein powder dissolved in water, known as a clear protein shake, during the first 6 months after bariatric surgery on fat-free mass loss in the first 12 months after LRYGB. The study design is a randomized, placebo-controlled, superiority trial that compares standard care (dietary recommendations alone) to additional protein drinks during the first 6 months postoperative (dietary recommendations plus additional protein drinks).

## Methods and analysis

### Study design

This is a double-blind randomized placebo-controlled trial to assess the effect of protein powder supplementation on fat-free mass loss in bariatric patients. The study was designed following the SPIRIT guidelines (Supplementary documents). This study will be conducted at the bariatric expertise center for obesity of the Maasstad Hospital Rotterdam, the sponsor. The sponsor will facilitate the study activities in the Maasstad Hospital Rotterdam. The sponsor does not have a role in the study design, collection, management, analysis, and interpretation of data, nor in writing the report or the decision to submit the report for publication. The sponsor does, however, have ultimate authority over all study activities. The funding party has no role in any of these activities. Executive investigators oversee the trial and take care of all administrative tasks associated with the trial. All data will be collected in “Castor Electronic Data Capture”, a data management system. This electronic file is accessible to the principal investigator and both executive investigators. Data will initially be collected on data paper-based entry forms which are anonymized and thus only contain participants’ study numbers. The forms will be kept in folders at the outpatient clinic in a locked file cabinet. Data will be entered into Castor by the executive investigators. Both researchers will check entered data by reviewing a random sample of study files entered by the other researcher. This will be done after the completion of every 50 files in Castor.

### Operation procedure and follow-up

All surgical procedures are performed by experienced bariatric surgeons. First, a gastric pouch of 25 cc is created. A 50-cm biliopancreatic limb is measured and the gastrojejunostomy is created using an endostapler and a continuous, absorbable suture. A side-to-side jejunojejunostomy is created using an endostapler and a continuous, absorbable suture, with an alimentary limb of 150 cm.

Afterwards, a transsection between both anastomoses of the jejunum is performed.

If the surgery is uncomplicated, patients are discharged from the hospital after one or two nights. Patients attend the outpatient clinic frequently during the first 5 years after surgery. During these visits, patients are assessed by the various members of the multidisciplinary bariatric team, e.g., surgeons, medical doctors, nurses, dieticians, and psychologists. Also, all patients are referred to the “movement program” unless physical activity is already well integrated in their pre-operative life. This program is a 10-week course of supervised physical training twice a week. Referrals are equal for both study groups. Blood samples are analyzed at multiple time points to screen for deficiencies or other abnormalities.

Study assessments take place during regular follow-up at the outpatient clinic. No additional visits are required.

### Intervention and control

Patients allocated to the intervention group will be asked to consume a clear protein powder shake containing 20 g of whey protein per serving daily dissolved in 200 mL of water during the first 6 months after surgery. Patients allocated to the control group will be asked to consume a clear placebo shake daily during the first 6 months after surgery. The placebo shake contains maltodextrine and is isocaloric with the protein shake. The placebo shake looks the same and has the same smell and taste. Both protein and maltodextrine are provided as a powder in single-use sachets. Study participants will dissolve the content of one sachet in 200 mL water and shake it until the powder is fully dissolved. All sachets look similarly neutral but contain a unique code. The codes are listed in the key document, indicating the content to be protein or placebo. Thus study participants are also blinded to the treatment. The allocated intervention will never be modified for a given trial participant. Discontinuing the intervention may be done at participants’ request or if unblinding is necessary due to a suspected allergic reaction to the study product. However, apart from the unlikely event of an allergic reaction, there are no anticipated harms in this low-risk trial.

Product specification sheets are enclosed with this manuscript as Supplementary documents.

### Study endpoints

#### Primary endpoint

The main study endpoint is the percentage fat-free mass loss at 6 months defined as fat-free mass loss (kg) divided by total weight loss (kg) × 100%. Fat-free mass will be assessed by multi-frequency bio-electrical impedance analysis (MF-BIA) using a Seca® MBCA 515. MF-BIA is an easy and non-invasive measurement tool and has been solidly validated for morbidly obese patients [10, 11]. BIA measurements will be conducted under standardized circumstances. Patients can wear light clothes, have to empty their pockets, and have to have an empty bladder. Patients will be asked to refrain from intensive physical activity, and intake of food and fluid 2 h prior to the measurements to minimize bias.

#### Secondary endpoints

Secondary endpoints are total weight loss, fat mass loss, BMI, hand grip strength, total protein intake, and attribution of dietary protein intake to total protein intake, measured at baseline and 1, 3, 6, and 12 months of follow-up.

Weight will be measured to the nearest 0.1 kg using the Seca® MBCA 515. Weight will be measured with light clothes on and without shoes and jackets. Height will be measured to the nearest 0.1 cm using a calibrated stadiometer. Height will be measured without shoes and patients will have to stand straight, with the heels against the wall and the face looking straight ahead.

Fat mass will be measured in kilograms (kg) and will be assessed by the Seca® MBCA 515 in the same way as fat-free mass. Hand grip strength will be measured using a grip strength dynamometer. Patients will be asked to remove jewelry from their fingers and the procedure will be explained to patients. Patients will be instructed to sit down and to hold the arm at a 90-degree angle. The measurement will be repeated three times on both sides and patients are allowed to rest for a minute in between the measurements. The highest score will be used for analysis.

Dietary protein intake will be assessed by using a 3-day food diary at baseline and on postoperative months 1, 3, 6, and 12. This method was chosen as it minimalizes recall bias and takes variations between week- and weekend days into account [12]. Patients will be asked to fill out the food diary for 2 weekdays and one weekend day in the week prior to the measurements. Patients will receive clear instructions to fill out the diary as adequately as possible. Patients will be asked to fill out exact quantities based on household sizes, including a slice of bread, a glass of milk or a bowl of yogurt. Patients will have to measure the content of the tableware they use in milliliters or grams to determine the food intake as accurately as possible. During the measurements, the food diary will be checked for completeness by the researchers. Any uncertainties will be discussed with the patient. If necessary, a book containing pictures of portion sizes for different foods and different household sizes of glasses, bowls, cutlery et cetera can be used. Protein intake will be estimated using the Dutch Food Composition Table (V. 2016, NEVO, RIVM, Bilthoven). If a product is not in the Dutch Food Composition Table, the energy- and protein content as indicated by the manufacturer will be used. Protein intake will be calculated to the nearest 0.1 g.

### Other study parameters

Other study parameters are baseline characteristics age, gender and ethnicity, and compliance and patient satisfaction regarding the shakes. Baseline characteristics will be obtained from the medical record. Compliance will be tracked by filling out a calendar in which patients can indicate whether they were able to consume the shake (partly or completely) every day. Patient satisfaction will be monitored by using a short questionnaire about the taste of the shakes.

Questionnaires are enclosed with this manuscript as Supplementary documents.

Physical activity will be assessed using the International Physical Activity Questionnaire (IPAQ) - Short [13]. This questionnaire focuses on physical activity in three domains, namely work activity, sports activity, and leisure activity. Activities are scored on a 1–5 scale, the higher the score the more intense the activity. The total score will be the sum of the scores on all domains.

All assessed endpoints per time point are depicted in Table 1.

**Table 1:** Study endpoints per time point

**Visit**	**Assessed study endpoints**
Before surgery (T0) (admission consultation with the surgeon)	*- Body height, age, gender* *- Body composition (body weight, fat-free mass, fat mass)* *- Hand grip strength* *- 3-day food diary* *- Questionnaire regarding physical activity* *- Questionnaire regarding participants’ attitude and compliance towards shake usage*
1 month after surgery (T1)	*- Body composition (body weight, fat-free mass, fat mass)* *- Hand grip strength* *- 3-day food diary* *- Questionnaire regarding physical activity* *- Questionnaire regarding participants’ attitude and compliance towards shake usage*
3 months after surgery (T2)	*- Body composition (body weight, fat-free mass, fat mass)* *- Hand grip strength* *- 3-day food diary* *- Questionnaire regarding physical activity* *- Questionnaire regarding participants’ attitude and compliance towards shake usage*
6 months after surgery (T3)	*- Body composition (body weight, fat-free mass, fat mass)* *- Hand grip strength* *- 3-day food diary* *- Study product calendar* *- Questionnaire regarding physical activity* *- Questionnaire regarding participants’ attitude and compliance towards shake usage*
12 months after surgery (T4)	*- Body composition (body weight, fat-free mass, fat mass)* *- Hand grip strength* *- 3-day food diary* *- Questionnaire regarding physical activity*

### Study population

Study participants are recruited in the bariatric expertise center for obesity of the Maasstad Hospital, Rotterdam, The Netherlands. Patients enrolled in this study will undergo a laparoscopic Roux-en-Y Gastric Bypass (LRYGB) at The Netherlands. Annually, 700 LRYGB procedures are performed in this center.

Patients are referred to the expertise center by either the general practitioner or a medical specialist. After referral, patients undergo a screening procedure to assess whether they are eligible for bariatric surgery according to the criteria of the International Federation for the Surgery of Obesity and Metabolic Disorders (IFSO).

The IFSO criteria are:

- Age 18–65 years
- BMI ≥ 40 kg/m2 by itself or BMI ≥ 35 kg/m^2^ with the presence of severe comorbidity related to morbid obesity
- Reasonable attempts at other weight loss techniques
- Obesity-related health problems
- No psychiatric or drug dependency problems
- A capacity to understand the risks and commitment associated with the surgery
- Pregnancy was not anticipated in the first 2 years following surgery

If necessary, psychological or dietary assessment and treatment will be performed before patients are accepted for surgery. If patients are eligible for bariatric surgery, informed consent will be given and patients will be scheduled for a LRYGB.

### Inclusion and exclusion criteria

In order to be eligible to participate in this study, patients will have to meet the IFSO criteria. Also, written informed consent must be obtained.

Patients will be excluded from participation in this study, when they meet one of the following criteria:

- Revisional bariatric surgery
- A protein-restricted diet for medical reasons
- Diagnosis of a (neuro-) muscular disease
- Inability to undergo MF-BIA (i.e., pregnancy, pacemaker)
- Allergy to any of the ingredients of either the protein or the placebo shake

### Inclusion

Patients who meet the inclusion criteria will be asked to participate in the study during the first visit to the bariatric expertise center for obesity, e.g., before they undergo bariatric surgery. Written informed consent will be obtained. Enrollment is done by one of the executive researchers. Personal information of enrolled participants is collected and stored in the key document. This document is locked and accessible only to executive researchers and principal investigator.

### Randomization

When enrolled in the study, study participants will be allocated to one of the two study groups, e.g., the protein group or the placebo group. Implementing of allocation is performed by handing out a package with powder sachets, either protein or placebo. The researcher handing out the package is blinded and thus does not know what is handed out. All packages contain enough one-portion sachets for one study participant during the full study period of 6 months. The packages are marked by the producer with a random batch number. These numbers contain a letter (A–Z) and three numbers (0–9). The batch numbers are computer-generated, unique, and randomly ordered. All batch numbers correspond to either protein or placebo, which is captured in a document. The document is not opened by the researchers unless unblinding is necessary. All packages that will be used during the full study period are produced at once. Fifty percent is protein, and 50% is placebo. The packages are randomly distributed on pallets from which they are handed out by the researcher. Thus, no block randomization is performed.

### Blinding

All study participants, researchers, and other care providers involved with study participants are blinded during the assessment of study outcomes, including the researcher performing the measurements. After completion of data collection, researchers will have access to the key document to unblind the results. Data analysts who will analyze the collected data after the study is finished, will not be blinded.

### Study timeline

First study assessments are done at the admission consultation with the surgeon, approximately 3 months before surgery. The final study assessment is done 12 months after surgery.

**Fig. 1:**
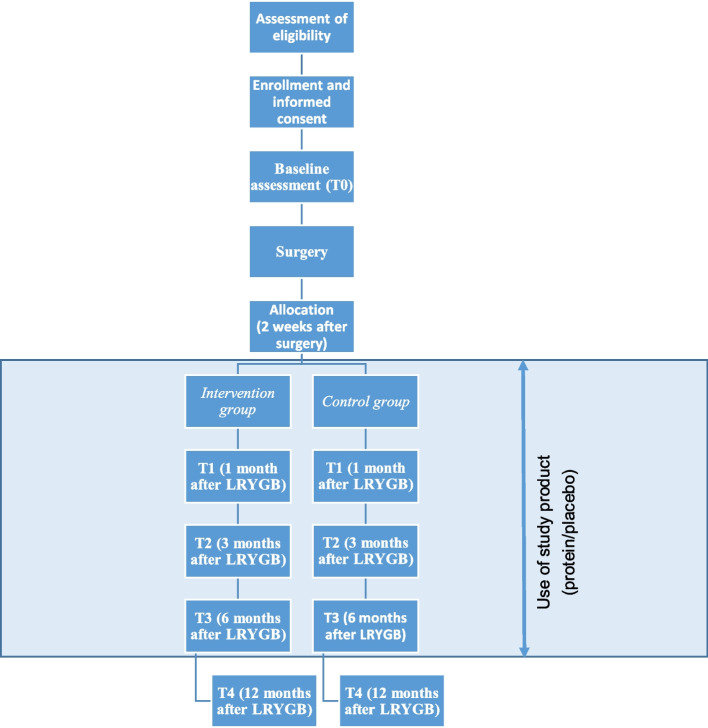
Study timeline PROMISE study

**Fig. 2:**
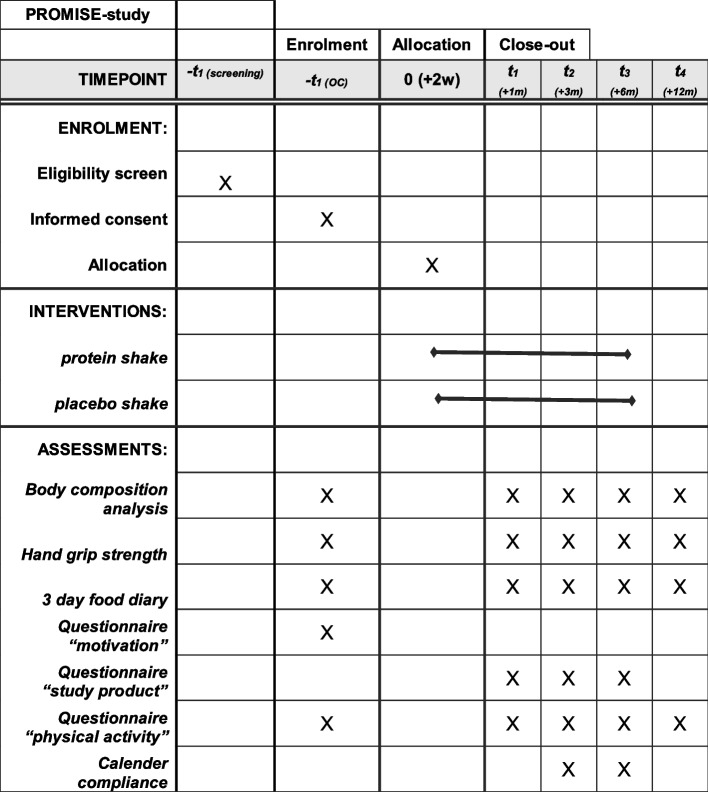
SPIRIT Figure

The complete study timeline is depicted in Figs. 1 and 2.

### Sample size calculation

Since similar studies on this topic are scarce, limited information is available on the expected effect size of the primary outcome, as well as its standard deviation, making a reliable sample size calculation challenging. Therefore, we will use a two-stage adaptive design recently proposed by Van Lancker et al. [14] that allows for a sample size re-assessment at a pre-specified point during the trial. Under this adaptive design, adequate control of the type 1 error rate in the effect estimate of the primary outcome can be ensured by using the adaptive *P*-value combination test to combine the *Z*-statistics obtained at the end of both stages [15, 16].

#### Initial sample size calculation

The following statistics were obtained from a prospective cohort study previously published on this topic [12].

**Table:** 

	Protein intake < 60 g/day	Protein intake ≥ 60 g/day
FFM loss/weight loss (%)		
6 months	25.5 ± 8.0 [*n* = 52]	20.0 ± 12.8 [*n* = 25]
12 months	21.5 ± 8.4 [*n* = 41]	23.1 ± 9.6 [*n* = 27]

Based on these statistics, the following total sample sizes required, using a two-sided independent *t*-test, alpha level of 5%, 1:1 allocation ratio, and assuming a common standard deviation of 10% for both groups, were obtained:

**Table:** 

Effect size	80% power	90% power
−5%	*n* = 128	*n* = 172
−4%	*n* = 200	***n* = 266**
−3%	*n* = 352	*n* = 470

#### Sample size re-assessment

A sample size of *n* = 266, enabling to detect a 4% difference with 90% power, is chosen as the initial target sample size. When *n* = 100 patients have reached their primary end-point at 6 months, a sample size re-assessment will be performed following the interim decision procedure proposed by Van Lancker et al. [14]. Sample size re-assessment is done based on the observed effect of conditional power. The weight for the first stage test statistic in the *p*-value combination test (and to re-assess the sample size) is set to *w* = 0.38, which corresponds with the information fraction of the (unadjusted) estimator that only uses the primary endpoint measurements (i.e., 100/266). Baseline covariates age, sex, and physical activity, as well as the intermediate outcome at 3 months, will be used to predict the treatment effect at the primary endpoint. The final sample size required to estimate the primary endpoint with a power of 90% (corresponding with an effect size of 4%) will be calculated. For practical reasons, the maximum number of patients we deem feasible to include within the planned timeframe is *n* = 500. In case the required number of patients exceeds this number, *n* = 500 patients will be included and a lower power will be accepted. In case the number of required patients is lower than the number of patients that have already been included at the time of the interim analysis, inclusion will be stopped. Upon re-assessment, the final sample size and estimated power will be communicated to the medical ethics committee as soon as possible, within 2 weeks.

#### Data Monitoring Committee (DMC)

No DMC has been established for this trial because it involves a study of short duration without critical safety concerns.

### Statistical analysis

A description of the planned statistical analyses for the outcomes of this study conform the ‘estimands’ framework [17] is presented in Tables 1 and 2 in the Supplementary material.

#### Primary study parameter(s)

The treatment effect for the primary outcome, i.e., the difference in the percentage of fat-free mass loss at 6 months between the intervention group and the control group, will be estimated using the adaptive* P*-value combination test [15, 16]. The combination test will be used to combine the Z-statistics of the treatment effect obtained at both stages of the trial (interim analysis and final analysis) and ensure adequate control of the type 1 error rate under this adaptive design. The analysis will be conducted according to the intention-to-treat principle. Since the measurement of the primary outcome coincides with a regular visit of standard care, the missing rate is expected to be low. If missings in the primary outcome do occur, these will be completed by imputation. The imputation model will include baseline covariates age, sex, physical activity, the intermediate outcome at 3 months, the treatment group, and all interactions with the treatment group.

#### Secondary study parameters

Secondary outcomes include percentage of fat-free mass loss, total weight loss, BMI, hand grip strength, and total protein intake measured at baseline, 3, 6, and 12 months of follow-up. These will be analyzed using a generalized least squares model (a linear model for longitudinal data). An unstructured correlation structure for the repeated measurements over time within each subject will be assumed. For each outcome, the treatment effect for the follow-up visits will be modeled by including covariates for treatment, visit, and its interactions. Missings in the outcome vector will be automatically accounted for by the longitudinal model, which allows subjects to remain in the analysis, as long as the outcome has been measured at least at one occasion. Unbiased estimates can still be obtained under the missing at random (MAR) assumption. The analyses will be conducted according to the intention-to-treat principle. As a secondary analysis, a per-protocol analysis will be conducted including only those patients in the treatment group with a cumulative intake of protein supplement of at least 70% during the 6-month intervention period, as indicated by the calendars. Missings in the calendar will be considered as no intake. In the placebo group, all patients will be included regardless of their level of compliance.

#### Other study parameters

The relationship between physical activity and the percentage of FFM loss after 3, 6, and 12 months will be assessed as follows. For each time point, a linear regression model will be fitted with the percentage of FFM loss as an outcome. As a measure for the cumulative physical activity during the study period, an area under the curve of the total scores of the IPAQ-SF up to the time point under consideration will be calculated. The AUC measure, as well as the indicator variable for treatment allocation and its interaction, will be included as covariates in the regression model. Missing covariates (IPAQ-SF) will be completed by multiple imputation using chained equations (MICE) [18].

Compliance with the protein intake will be evaluated by analysis of the diaries. For each patient cumulative intake will be calculated and compared between the intervention and placebo groups by simple descriptive statistics. In addition, trends in compliance over time will be explored and presented by descriptive statistics and/or visualized by means of for instance bar charts or box plots.

#### Interim analysis

As described in the “Sample size re-assessment” section an interim analysis for sample size re-assessment will be performed at the moment *n* = 100 patients have reached their primary endpoint at 6 months. The interim analysis will be conducted by a statistician of the Maasstad Hospital (Martijn Kuijper) in collaboration with investigators of this study (Annick Taselaar, PhD candidate, and Joanne Boes, dietician). Furthermore, Kelly Van Lancker, statistician and author of the sample size re-assessment method [14], is involved with this project and will be available for advice and assistance with the interim analysis.

### Retention of study participants

All study measurements are done during regular follow-up visits to enhance compliance to the protocol. The team involved in this study was trained to motivate participants to adhere to the study protocol. Different flavors of the study product are included to enhance compliance. All participants receive more drinks than needed for the follow-up time which allows participants to choose their favorite flavor and leave out the flavors they like less.

When the study product is not tolerated well, the researcher will discuss possible solutions with the individual study participant. Examples of possible solutions are:

- Spread the drink throughout the day
- Dissolve the drink in extra water
- Skip intake of the study product for several days and then try again

If despite the above advice participant does not tolerate the drinks, intake may be stopped. Participant does not need to withdraw from the study, since compliance to the product is one of the study parameters. These patients won’t be taken into account in the per-protocol analysis if their compliance is below 70% (see the “Secondary study parameters” section on page 13). All other measurements will be continued in this situation. Also, the lack of intake is documented in the calendar. Since the study is double-blinded, the allocation of intervention will never be modified.

#### Withdrawal from the study

Participants may withdraw from the study at any time point on their own request. Participants have stated that data collected until withdrawal can be used for analysis.

#### Protocol modifications

If any modifications to the protocol are made, these are submitted to the medical ethical committee for approval. This will be done by a protocol amendment. Once the amendment is approved, it will be communicated to the team involved in the study verbally, by e-mail, and by modification of the study protocol which is available at the outpatient clinic. Other relevant parties such as trial registries and journals will be informed by e-mail. If the modification is relevant for previously included participants, they will be informed verbally by phone or during their visit to the outpatient clinic.

## Trial status

The protocol, version number 2, dated 20th of February 2022, was approved by the ethical committee on 10th of May 2022. Recruitment started on the 1st of September 2022. Enrolment of the first participant was on September 16^th^, 2022. Currently, recruitment is ongoing. Recruitment will be completed by approximately August 2023.

## Ethics and dissemination

### Ethical approval and monitoring

Ethical approval was given by the Medical Research Ethics Committees United (MEC-U) (NL 80414.100.22). Monitoring is done by an external, qualified monitor.

### Risks and reporting of (serious) adverse events

#### Temporary halt for reasons of subject safety

In accordance with section 10, subsection 4, of the Dutch law on medical scientific research “Wet medisch-wetenschappelijk onderzoek met mensen” (WMO), the sponsor will suspend the study if there is sufficient ground that continuation of the study will jeopardize the subject health or safety. The sponsor will notify the accredited METC without undue delay of a temporary halt including the reason for such an action. The study will be suspended pending a further positive decision by the accredited METC. The investigator will take care that all subjects are kept informed.

#### Adverse events (AEs) and serious adverse events (SAEs)

All adverse events reported spontaneously by the subject or observed by the investigator or his staff will be recorded.

The investigator will report all SAEs to the sponsor without undue delay after obtaining knowledge of the events. The sponsor will report the SAEs through the Dutch web portal ToetsingOnline to the accredited METC that approved the protocol (MEC-U), within 7 days of first knowledge for SAEs that result in death or are life-threatening followed by a period of a maximum of 8 days to complete the initial preliminary report. All other SAEs will be reported within a period of a maximum of 15 days after the sponsor has first knowledge of the serious adverse events.

#### Follow-up of adverse events

All AEs will be followed until they have abated, or until a stable situation has been reached. Depending on the event, follow-up may require additional tests or medical procedures as indicated, and/or referral to the general physician or a medical specialist. SAEs need to be reported till end of the study within the Netherlands, as defined in the protocol.

### Access to the final trial dataset

The final dataset will be available to the principal investigator and both executive investigators. Access is connected to the employment agreement and thus will be eliminated once the contract is terminated.

### Dissemination of study results

Results of this study will be submitted to peer-reviewed journals and will be presented at national and international conferences.

### IPD sharing statement

Access to trial IPD can be requested by qualified researchers engaging in independent scientific research and will be provided following review and approval of a research proposal and Statistical Analysis Plan (SAP) and execution of a Data Sharing Agreement (DSA). For more information or to submit a request, please contact wetenschapsbureau@maasstadziekenhuis.nl.

## Discussion

This study will be the first RCT specifically designed to assess the effect of protein supplementation in the form of a clear protein powder shake after bariatric surgery, more specifically after laparoscopic Roux-en-Y gastric bypass (RYGB). The primary endpoint is the percentage of fat-free mass loss after 6 months. This is defined as the fat-free mass loss as a percentage of total weight loss.

This study is conducted in a highly specialized center for bariatric surgery. Study assessments are done by an experienced team consisting of bariatric surgeons, medical doctors, physical assistants, nurse practitioners, dieticians, and psychologists.

The study is diligently designed to supply valuable data for this patient group. However, some challenges are faced inherent to this patient group. Like most clinical trials, the applicability of study results relies on the compliance of study participants.

Reiber et al. found that many bariatric patients are non-compliant to follow-up after bariatric surgery [19]. 217 of the included 398 patients (55%) in their study were identified as non-compliant because they missed the scheduled appointment and at least one appointment overall.

This non-compliance to follow-up may result in dropout of participants in our study as well. However, the center conducting the study has shown to be able to keep patients in follow-up after bariatric surgery very well, especially the first years, according to annual auditing data (DATO). The low number of patients lost to follow-up after surgery in our center is achieved by a periodic check of all patients in follow-up and their future appointments. If this check shows that patients have missed or canceled their appointments, they are being actively approached to schedule new visits to the hospital. This is standard practice in our center in order to minimize the amount of patients lost to follow-up after surgery.

A recent study by Steenackers et al. assessed compliance to multivitamin supplementation after bariatric surgery. They found that only 24.9% of all patients reported high compliance whereas 17.8% did not take any multivitamins at all, despite extensive recommendations on this subject [20].

Smelt et al. also found that adherence to multivitamins after bariatric surgery was poor. They identified several potential causes for it of which the most important were eating behavior, postoperative complications leading to gastrointestinal symptoms, treatment complexity, composition of multivitamins, and costs of multivitamin treatment [21].

To reduce the influence of poor compliance, some precautions have been taken in the design of this study. All patients are screened before surgery to examine their eating behavior. If there are deficiencies in these behaviors, such as lack of regularity or too low an eating frequency, for example, patients are referred to a preparatory course with a dietician before surgery is scheduled. To lower treatment complexity, the use of a daily shake was chosen instead of one that must be used more often in a day. Furthermore, the shakes are packaged per portion and participants will receive a special shaker bottle which makes it easy to prepare the shake. Specifically, a clear shake is used instead of a milk-based shake because the composition of a clear shake is thought to be tolerated better after bariatric surgery because of changed food preferences [9].

Costs will have no bearing on the compliance since study products are provided for free. The potential consequence of costs must however be a concern gets the supplementation gets implemented in future standard postoperative care.

The team is trained to motivate study participants to be compliant to the shakes. Nonetheless, lack of compliance despite this motivational counseling, might be valuable information as well. Therefore, compliance is investigated using a calendar to monitor the compliance. Study participants will write down whether they used the study product that day fully, partly, or not at all. Study participants are encouraged to fill in this calendar honestly, rather than socially desirable. Next to the calendar, questionnaires are conducted to investigate reasons for (in)compliance.

Paradis et al. wrote about bias in surgical trials and the effect it has on the internal and external validity of research [22]. This research protocol was designed in a way that bias is reduced as much as possible, mostly by randomization and blinding of both the researchers and the study participants. Some aspects, however, must be considered potential risk for bias as described in the paper by Paradis.

First of all, the risk of sampling bias must be reviewed because it may affect the generalizability of the results and thus external validity. This form of bias might occur when patients who are relatively active, are more prone to participate in the study because they are more interested in their own muscle mass. Physical activity is considered a potential confounder because it affects muscle mass in obese adults as described by Willis et al. [23], although this effect has not been found in the bariatric surgery population [24]. To reduce the effect of this potential bias, the level of physical activity of study participants is measured by validated questionnaires at every time point. Results can be stratified for different levels of activity to correct for this potential confounder.

Interpatient variability in compliance to the postoperative protocol could also result in sampling bias. More compliant participants might also follow the dietary counseling for high-protein food products more carefully than less compliant participants. This might lead to a higher total protein intake, not necessarily as a result of the additional protein shake. Hence, results must also be corrected for total dietary protein intake. This will be measured by a 3-day food diary at the time of body composition assessments. This method was chosen as it minimalizes recall bias and takes variations between week- and weekend days into account [12].

Another risk of bias, more specifically attrition bias, is arising from the use of a placebo in the control group. In this study, maltodextrine is used as the main ingredient to create an isocaloric alternative for the protein drink. Maltodextrine itself could affect body composition and in that way become a confounder. Other nutrients that could be used to add calories are fat or alcohol. However, fat is not easily soluble in a clear shake and alcohol is obviously undesirable from the medical perspective. For this reason, maltodextrine is considered the best comparator in this study design.

Lastly, transfer bias might occur when the protein drink or the placebo is tolerated better than the other product leading to excessive dropout in one of the study groups. To minimize this form of bias, the team is trained to encourage patients’ compliance to follow-up.

The study design is adapted to these calculated risks of bias. Therefore, reliable and generalizable results are expected. The results will be published and presented at international congresses and might hopefully lead to further research and thus improvement of care of bariatric surgery patients.

### Supplementary Information

**Additional file 1.**

**Additional file 2.**

**Additional file 3: Table 1a.** Primary outcome: Percentage of fat free mass loss at 6 months post-surgery (intention-to-treat). **Table 1b.** Secondary outcome: Percentage of fat free mass loss at 6 months post-surgery (per protocol). **Table 2a.** Primary outcome: Percentage of fat free mass loss at 1, 3, (6) and 12 months post-surgery (intention-to-treat).^§^**Table 2b.** Secondary outcome: Percentage of fat free mass loss at 1, 3, (6) and 12 months post-surgery (per protocol).^§^

**Additional file 4.** SPIRIT 2013 Checklist: Recommended items to address in a clinical trial protocol and related documents.

## Data Availability

Not applicable.

## References

[1] Lupoli R, Lembo E, Saldalamacchia G, Avola CK, Angrisani L, Capaldo B (2017). Bariatric surgery and long-term nutritional issues. World J Diabetes.

[2] Martinez MC, Meli EF, Candia FP, Filippi F, Vilallonga R, Cordero E (2022). The impact of bariatric surgery on the muscle mass in patients with obesity: 2-year follow-up. Obes Surg.

[3] Oppert JM, Bellicha A, Roda C, Bouillot JL, Torcivia A, Clement K (2018). Resistance training and protein supplementation increase strength after bariatric surgery: a randomized controlled trial. Obesity (Silver Spring).

[4] Abdulsalam F, Ali HI, Altinoz A, Nimeri A (2021). The effect of protein consumption on fat-free mass, fat mass, and weight loss 1 year after sleeve gastrectomy and Roux-en-Y gastric bypass. Obes Surg.

[5] Nuijten MAH, Monpellier VM, Eijsvogels TMH, Janssen IMC, Hazebroek EJ, Hopman MTE (2020). Rate and determinants of excessive fat-free mass loss after bariatric surgery. Obes Surg.

[6] Wolfe RR (2006). The underappreciated role of muscle in health and disease. Am J Clin Nutr.

[7] Schollenberger AE, Karschin J, Meile T, Kuper MA, Konigsrainer A, Bischoff SC (2016). Impact of protein supplementation after bariatric surgery: a randomized controlled double-blind pilot study. Nutrition.

[8] Odstrcil EA, Martinez JG, Santa Ana CA, Xue B, Schneider RE, Steffer KJ (2010). The contribution of malabsorption to the reduction in net energy absorption after long-limb Roux-en-Y gastric bypass. Am J Clin Nutr.

[9] Guyot E, Dougkas A, Robert M, Nazare JA, Iceta S, Disse E (2021). Food preferences and their perceived changes before and after bariatric surgery: a cross-sectional study. Obes Surg.

[10] de Oliveira PAP, Montenegro ACP, Bezerra LRA, da Conceicao Chaves de Lemos M, Bandeira F (2020). Body composition, serum sclerostin and physical function after bariatric surgery: performance of dual-energy X-ray absorptiometry and multifrequency bioelectrical impedance analysis. Obes Surg.

[11] Beato GC, Ravelli MN, Crisp AH, de Oliveira MRM (2019). Agreement between body composition assessed by bioelectrical impedance analysis and doubly labeled water in obese women submitted to bariatric surgery : body composition, BIA, and DLW. Obes Surg.

[12] Shim JS, Oh K, Kim HC (2014). Dietary assessment methods in epidemiologic studies. Epidemiol Health.

[13] Craig CL, Marshall AL, Sjostrom M, Bauman AE, Booth ML, Ainsworth BE (2003). International physical activity questionnaire: 12-country reliability and validity. Med Sci Sports Exerc.

[14] Van Lancker K, Vandebosch A, Vansteelandt S (2020). Improving interim decisions in randomized trials by exploiting information on short-term endpoints and prognostic baseline covariates. Pharm Stat.

[15] Bauer P, Bretz F, Dragalin V, Konig F, Wassmer G (2016). Twenty-five years of confirmatory adaptive designs: opportunities and pitfalls. Stat Med.

[16] Bauer P, Kohne K (1996). Evaluation of experiments with adaptive interim analysis (vol 50, pg 1029, 1994). Biometrics.

[17] Kang M, Kendall MA, Ribaudo H, Tierney C, Zheng L, Smeaton L (2022). Incorporating estimands into clinical trial statistical analysis plans. Clin Trials.

[18] Azur MJ, Stuart EA, Frangakis C, Leaf PJ (2011). Multiple imputation by chained equations: what is it and how does it work?. Int J Methods Psychiatr Res.

[19] Reiber BMM, Leemeyer AR, Bremer MJM, de Brauw M, Bruin SC (2021). Weight loss results and compliance with follow-up after bariatric surgery. Obes Surg.

[20] Steenackers N, Vandewynckel S, Boedt T, Deleus E, Hoekx S, Lannoo M (2022). Compliance and patients’ perspectives towards nutritional supplementation following bariatric surgery. Obes Surg.

[21] Smelt HJM, Pouwels S, Smulders JF, Hazebroek EJ (2020). Patient adherence to multivitamin supplementation after bariatric surgery: a narrative review. J Nutr Sci.

[22] Paradis C (2008). Bias in surgical research. Ann Surg.

[23] Willis LH, Slentz CA, Bateman LA, Shields AT, Piner LW, Bales CW (2012). Effects of aerobic and/or resistance training on body mass and fat mass in overweight or obese adults. J Appl Physiol.

[24] Bellicha A, van Baak MA, Battista F, Beaulieu K, Blundell JE, Busetto L (2021). Effect of exercise training before and after bariatric surgery: a systematic review and meta-analysis. Obes Rev.

