# Supplementary material for: PROMISE: effect of protein supplementation on fat-free mass preservation after bariatric surgery, a randomized double-blind placebo-controlled trial
Source: Trials. 2023 Nov 9;24:717. doi: 10.1186/s13063-023-07654-w (PMC10636856; doi:10.1186/s13063-023-07654-w)
Supplement: Supplementary file 1 — Additional file 1. [file 13063_2023_7654_MOESM1_ESM.zip › Product specification sheet ProteinR1.pdf]

DRAFT Product Specification Sheet

CLEAR PROTEIN Apple Pear flavoured

FIT FOR ME

MDP-1-193-A

03/12/2021 Version: 1

Aminolabs
by Eurofood Belgium nv
Research Campus 6
B-3500 Hasselt
Belgium
T: +32 11 28 10 00
www.aminolabs.com

Ingredient list:

Whey protein isolate (**Milk**), Acidity regulators (Malic acid (E296) and - Citric acid (E330)), Flavour, Anti-foaming agent (vegetable oil (soy), carnauba wax (E903)), Sweetener (- Sucralose (E955)), Colour (- Turmeric (E100)).

Is produced in a facility that also handles sulphites, cereals containing gluten, crustaceans, eggs, fish, soybeans, milk, mustard and products derived from the above.

Serving

26g/200ml of water

Typical Nutritional Information[1]

	========= Per 100 g =========		========= Per 26 g =========		
					% RI
Energy (kJoule)	1492	kJ	388	kJ	
Energy (kCal)	351	kcal	91	kcal	5
Fat	0	g	0	g	0
Saturated fatty acids	0.1	g	0	g	0
Carbohydrates	2.5	g	0.7	g	0
Sugars	0	g	0	g	0
Fiber	0	g	0	g	
Protein (As is)	77	g	20	g	40
Salt	0.05	g	0.01	g	0

*[1] As foreseen in the FIC Regulation 1169/2011, Annex I (10), protein is calculated per 100g of product and/or per portion using the conversion factor for nitrogen*6.25.*
% Reference Intake based on the requirements of a 2000 kcal / 8400 kJ diet for adults

Please note that this document is preliminary and may be subject to change.
The values on this document are an estimation based on the calculations
of the specifications of the raw materials provided to us by our suppliers.

Aminolabs
by Eurofood Belgium nv
Research Campus 6
B-3500 Hasselt
Belgium
T: +32 11 28 10 00
www.aminolabs.com

Allergens²

Cereals containing gluten	-	Nuts	-
Crustaceans	-	Celery	-
Eggs	-	Mustard	-
Fish	-	Sesame seeds	-
Peanuts	-	Sulphur dioxide / sulphites	-
Soy	-	Lupin	-
Milk	+	Moluscs	-

+ : present
 - : absent

² *Based on specifications of raw materials provided to us by our suppliers.*

Storage & transport

Store and transport in original closed packaging in a dry and dark place at ambient temperature (between 15°C to 25°C), out of reach of young children.
