# Supplementary material for: PROMISE: effect of protein supplementation on fat-free mass preservation after bariatric surgery, a randomized double-blind placebo-controlled trial
Source: Trials. 2023 Nov 9;24:717. doi: 10.1186/s13063-023-07654-w (PMC10636856; doi:10.1186/s13063-023-07654-w)
Supplement: Supplementary file 2 — Additional file 2. [file 13063_2023_7654_MOESM2_ESM.zip › Questionnaire shake T3 en-GBR1.docx]

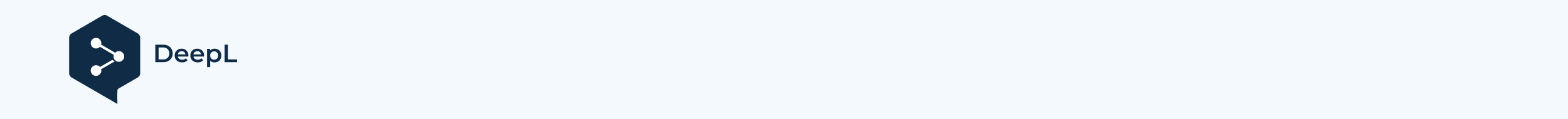


Subscribe to DeepL Pro to edit this document.
Visit [www.DeepL.com/pro](https://www.deepl.com/pro?cta=edit-document) for more information.

##
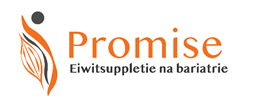
Study number________

## Date:______________

Questionnaire 2

You are taking part in the PROMISE study. This means that you have had gastric bypass surgery and now take an extra drink every day, in addition to your food and drink. During your hospital visits, measurements will be taken to determine your body composition, particularly the amount of fat mass and muscle mass.

With this study, we want to investigate whether patients who take a protein drink after their gastric bypass lose less muscle mass while losing weight. We would now like to ask you 3 questions.

# Question 1

What do you think of the drinks?

*(circle the most appropriate answer)*

**1** (very dirty) **2** (not so good) **3** (quite nice) **4 (**very tasty)

# Question 2

Are you managing to use the drink every day so far?

*(circle the most appropriate answer)*

**1** (not at all) **2** (hardly at all) **3** (a little) **4 (**very much)

# Question 3

What do you find difficult about using the drink?

- I find the taste gross
- I don't get the quantity on
- I forget to use the booze
- I am too tired to take the drink
- I feel too sick to take the drink
- Anders:________________________________________________________________
