# Supplementary material for: PROMISE: effect of protein supplementation on fat-free mass preservation after bariatric surgery, a randomized double-blind placebo-controlled trial
Source: Trials. 2023 Nov 9;24:717. doi: 10.1186/s13063-023-07654-w (PMC10636856; doi:10.1186/s13063-023-07654-w)
Supplement: Supplementary file 2 — Additional file 2. [file 13063_2023_7654_MOESM2_ESM.zip › Questionnaire regarding physical activity (IPAQ)R1.pdf]

INTERNATIONALE LICHAMELIJKE ACTIVITEITEN VRAGENLIJST

KORTE VERSIE

Wij zijn geïnteresseerd welke vorm(en) van lichamelijke activiteit mensen verrichten in hun dagelijkse leven. De vragen gaan over uw lichamelijke activiteit **gedurende de afgelopen 7 dagen**. Beantwoordt u alstublieft alle vragen, ook al beschouwt u uzelf als niet lichamelijk actief. Denkt u aan activiteiten die u doet op het werk, in en rond het huis, om van de ene naar de andere plaats te komen en activiteiten in uw vrije tijd voor recreatie, training of sport.

Denkt u aan alle zware lichamelijke activiteiten die u deed in **de afgelopen 7 dagen**. **Zware** lichamelijke activiteiten zijn activiteiten die veel lichamelijke inspanning kosten en voor een veel snellere ademhaling zorgen. Denk *alleen* aan de activiteiten die u ten minste 10 minuten per keer heeft verricht.

1. Als u denkt aan **de afgelopen 7 dagen**, op hoeveel van deze dagen heeft u **zware** lichamelijke activiteiten verricht zoals zware lasten tillen, spitten, aerobics of wielrennen?

_ **dagen per week**

☐ geen zware lichamelijke activiteiten → **ga naar vraag 3**

2. Op de dagen dat u zwaar lichamelijk actief was, hoeveel tijd heeft u daar dan gewoonlijk aan besteed?

_ **uren per dag**
_ **minuten per dag**

☐ Weet niet / niet zeker

Denkt u aan activiteiten die **matige** lichamelijke inspanning kosten en die u in **de afgelopen 7 dagen** heeft verricht. **Matig** intensieve lichamelijke activiteit laat u iets sneller ademen dan normaal. Denkt u weer alleen aan activiteiten die u ten minste 10 minuten per keer heeft verricht.

3. Als u denkt aan **de afgelopen 7 dagen**, op hoeveel van deze dagen heeft u **matig** intensieve lichamelijke activiteit verricht, zoals het dragen van lichte lasten, fietsen in een normaal tempo of dubbeltennis? Laat wandelen hier buiten beschouwing.

_ **dagen per week**

☐ geen matig lichamelijke activiteiten → **ga naar vraag 5**

4. Op de dagen dat u **matig** intensief lichamelijk actief was, hoeveel tijd heeft u daar dan gewoonlijk aan besteed?

_ **uren per dag**
_ **minuten per dag**

☐ Weet niet / niet zeker

5. Als u denkt aan **de afgelopen 7 dagen**, op hoeveel dagen heeft u tenminste 10 minuten per keer **gewandeld**? Denk hierbij aan wandelen op het werk en thuis, wandelen om van de ene naar de andere plaats te komen, en al het andere wandelen dat u deed tijdens recreatie, sport of vrijetijdsbesteding.

_ **dagen per week**

☐ geen wandelen → **ga naar vraag 7**

6. Op de dagen dat u ten minste 10 minuten per keer **wandelde**, hoeveel tijd heeft u daar dan gewoonlijk aan besteed?

_ **uren per dag**
_ **minuten per dag**

☐ Weet niet / niet zeker

7. Hoeveel tijd bracht u gewoonlijk **zittend** door gedurende een **doordeweekse** dag **in de afgelopen 7 dagen**? Bij deze tijd mag zitten achter een bureau, tijd die zittend wordt doorgebracht met vrienden, zittend lezen, studeren of tv kijken worden gerekend.

_ **uren per dag**
_ **minuten per dag**

☐ Weet niet / niet zeker

Dit is het einde van de vragenlijst. Dank voor uw medewerking.
