# Supplementary material for: PROMISE: effect of protein supplementation on fat-free mass preservation after bariatric surgery, a randomized double-blind placebo-controlled trial
Source: Trials. 2023 Nov 9;24:717. doi: 10.1186/s13063-023-07654-w (PMC10636856; doi:10.1186/s13063-023-07654-w)
Supplement: Supplementary file 2 — Additional file 2. [file 13063_2023_7654_MOESM2_ESM.zip › Questionnaire regarding shake usage T0R1.docx]

## Studienummer________


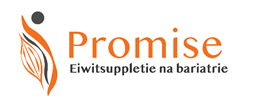


## Datum:______________

Vragenlijst 1

U neemt deel aan de PROMISE-study. Dat betekent dat u komende 6 maanden iedere dag een extra drank zult nemen, naast uw eten en drinken. Tijdens de bezoeken in het ziekenhuis zullen er metingen worden gedaan om uw lichaamssamenstelling te bepalen, met name de hoeveelheid vetmassa en spiermassa.

Met deze studie willen we onderzoeken of patiënten die na hun maagverkleining een eiwitdrank gebruiken, minder spiermassa verliezen tijdens het afvallen. We willen u nu 3 vragen stellen.

# Vraag 1

Denkt u zelf dat het zou kunnen helpen om een extra eiwitdrank te gebruiken?

*(omcirkel het best passende antwoord)*

**1** (helemaal niet) **2** (bijna niet) **3** (wel een beetje) **4** (heel erg)

# Vraag 2

Kijkt u ernaar uit om een extra drank te gebruiken?

*(omcirkel het best passende antwoord)*

**1** (helemaal niet) **2** (bijna niet) **3** (wel een beetje) **4** (heel erg)

# Vraag 3

Denkt u zelf dat het gaat lukken om de drank iedere dag te gebruiken?

*(omcirkel het best passende antwoord)*

**1** (helemaal niet) **2** (bijna niet) **3** (wel een beetje) **4** (heel erg)
