# Supplementary material for: PROMISE: effect of protein supplementation on fat-free mass preservation after bariatric surgery, a randomized double-blind placebo-controlled trial
Source: Trials. 2023 Nov 9;24:717. doi: 10.1186/s13063-023-07654-w (PMC10636856; doi:10.1186/s13063-023-07654-w)
Supplement: Supplementary file 2 — Additional file 2. [file 13063_2023_7654_MOESM2_ESM.zip › Questionnaire regarding shake usage T1, T2R1.docx]

##
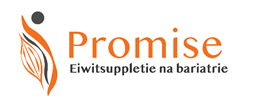
Studienummer________

## Datum:______________

Vragenlijst 2

U neemt deel aan de PROMISE-study. Dat betekent dat u een maagverkleining heeft ondergaan en nu iedere dag een extra drank neemt, naast uw eten en drinken. Tijdens de bezoeken in het ziekenhuis worden er metingen worden gedaan om uw lichaamssamenstelling te bepalen, met name de hoeveelheid vetmassa en spiermassa.

Met deze studie willen we onderzoeken of patiënten die na hun maagverkleining een eiwitdrank gebruiken, minder spiermassa verliezen tijdens het afvallen. We willen u nu 4 vragen stellen.

# Vraag 1

Wat vindt u van de dranken?

*(omcirkel het best passende antwoord)*

**1** (heel vies) **2** (niet zo lekker) **3** (best lekker) **4** (heel erg lekker)

# Vraag 2

Lukt het tot nu toe om de drank iedere dag te gebruiken?

*(omcirkel het best passende antwoord)*

**1** (helemaal niet) **2** (bijna niet) **3** (wel een beetje) **4** (heel erg)

# Vraag 3

Wat vindt u moeilijk aan het gebruiken van de drank?

- De smaak vind ik vies
- De hoeveelheid krijg ik niet op
- Ik vergeet de drank te gebruiken
- Ik ben te moe om de drank te gebruiken
- Ik voel me te ziek om de drank te gebruiken
- Anders:________________________________________________________________

# Vraag 4

Denkt u dat het de komende tijd gaat lukken om de drank iedere dag te gebruiken?

*(omcirkel het best passende antwoord)*

**1** (helemaal niet) **2** (bijna niet) **3** (wel een beetje) **4** (heel erg)
