# Supplementary material for: PROMISE: effect of protein supplementation on fat-free mass preservation after bariatric surgery, a randomized double-blind placebo-controlled trial
Source: Trials. 2023 Nov 9;24:717. doi: 10.1186/s13063-023-07654-w (PMC10636856; doi:10.1186/s13063-023-07654-w)
Supplement: Supplementary file 2 — Additional file 2. [file 13063_2023_7654_MOESM2_ESM.zip › Questionnaire shake T0 en-GB (1)R1.docx]

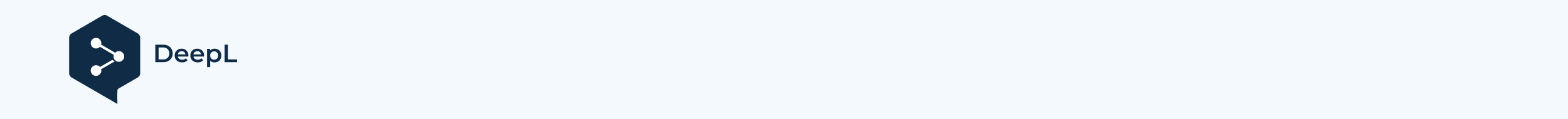


Subscribe to DeepL Pro to edit this document.
Visit [www.DeepL.com/pro](https://www.deepl.com/pro?cta=edit-document) for more information.

## Study number________


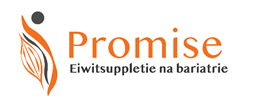


## Date:______________

Questionnaire 1

You are taking part in the PROMISE study. This means you will take an extra drink every day for the next 6 months, in addition to your food and drink. During visits to the hospital, measurements will be taken to determine your body composition, particularly the amount of fat mass and muscle mass.

With this study, we want to investigate whether patients who take a protein drink after their gastric bypass lose less muscle mass while losing weight. We would now like to ask you 3 questions.

# Question 1

Do you personally think it might help to consume an additional protein drink?

*(circle the most appropriate answer)*

**1** (not at all) **2** (hardly at all) **3** (a little) **4** (very much)

# Question 2

Looking forward to having an extra drink?

*(circle the most appropriate answer)*

**1** (not at all) **2** (hardly at all) **3** (a little) **4 (**very much)

# Question 3

Do you think you yourself will manage to consume the drink every day?

*(circle the most appropriate answer)*

**1** (not at all) **2** (hardly at all) **3** (a little) **4 (**very much)
