# Supplementary figures and images for: PROMISE: effect of protein supplementation on fat-free mass preservation after bariatric surgery, a randomized double-blind placebo-controlled trial
Source: Trials. 2023 Nov 9;24:717. doi: 10.1186/s13063-023-07654-w (PMC10636856; doi:10.1186/s13063-023-07654-w)

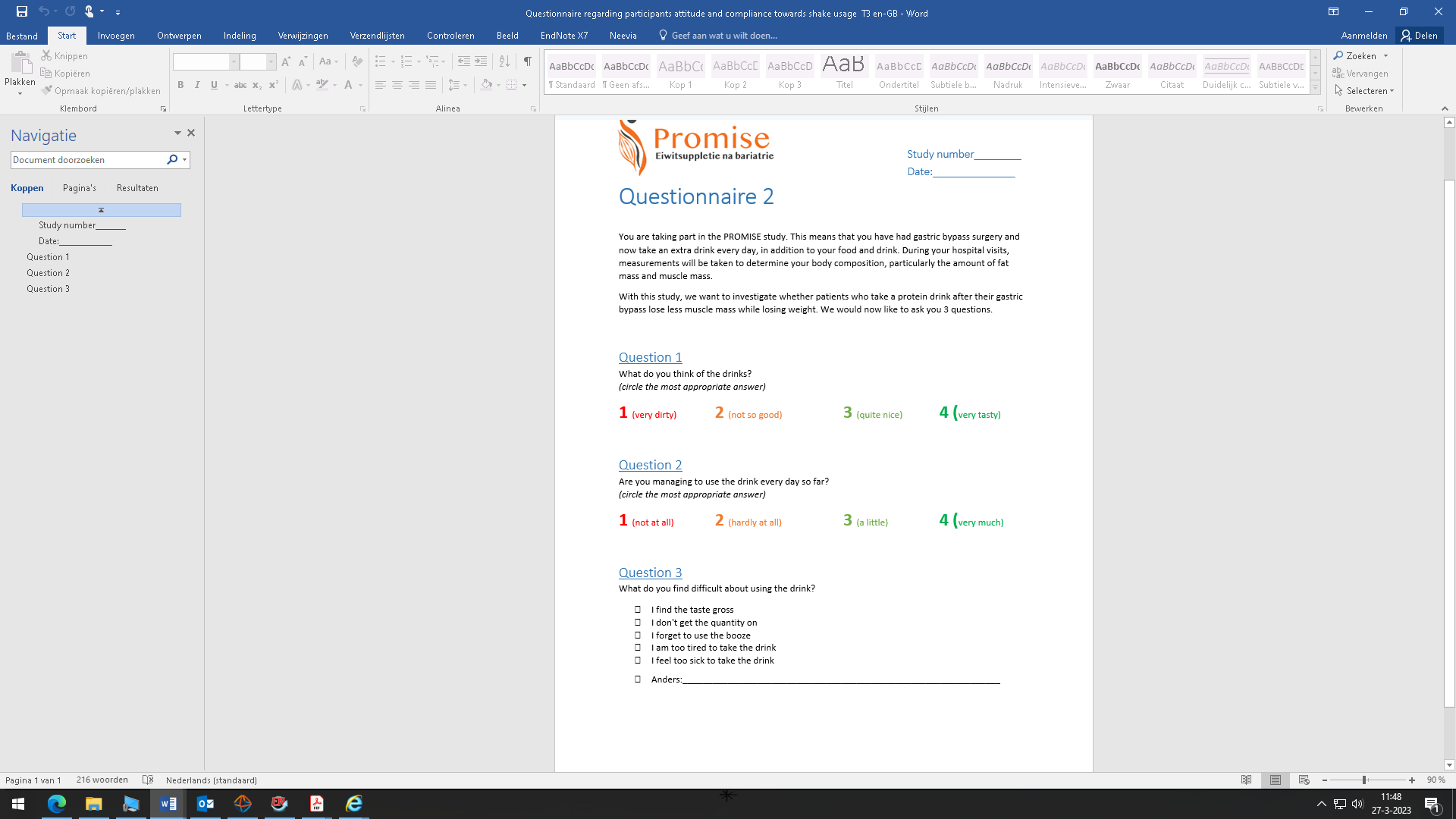


# **3**


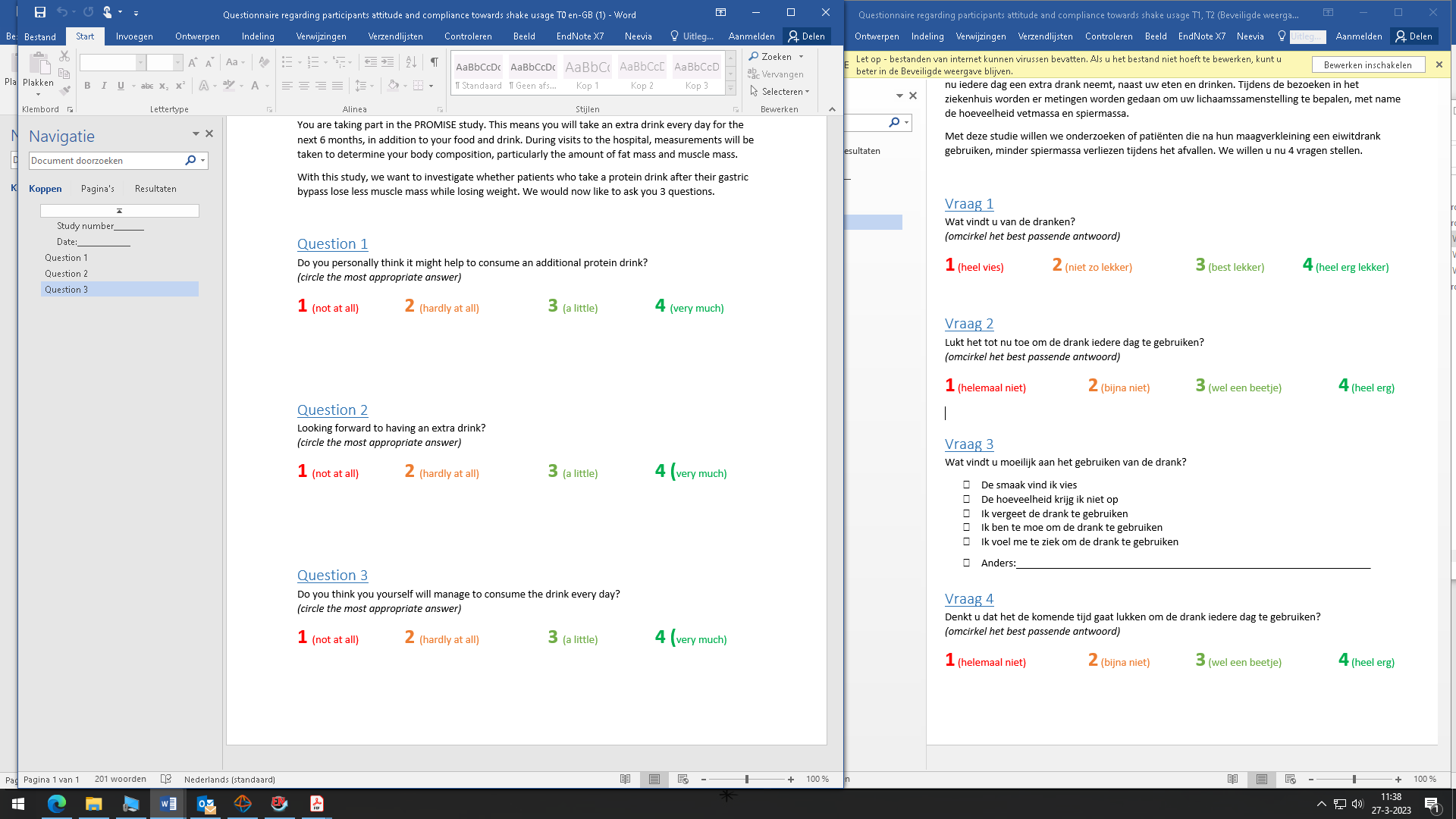

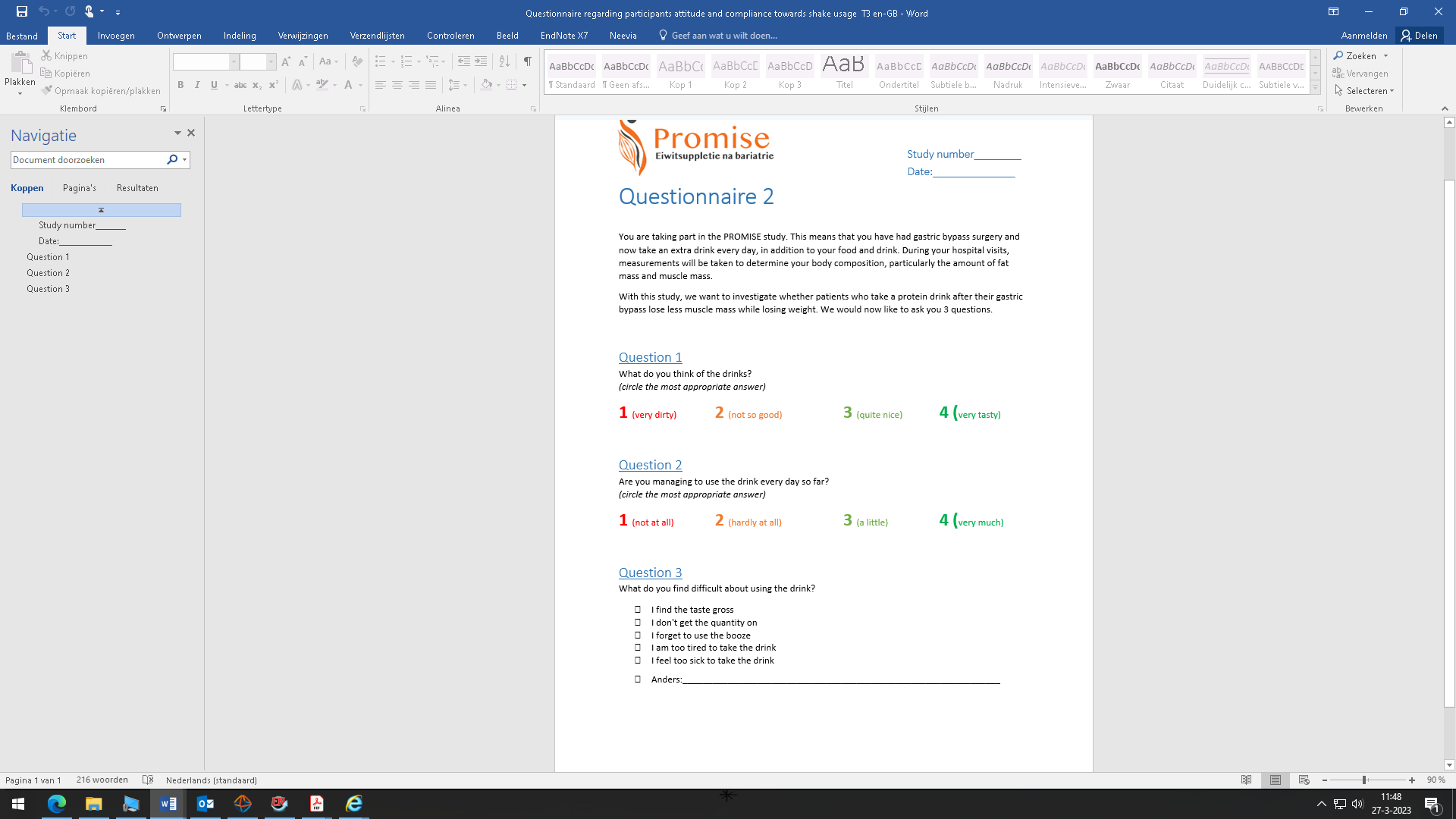


**This is a translation of the official document for the purpose of manuscript review**

# _4

Supplement: Supplementary file 2 — Additional file 2. [file 13063_2023_7654_MOESM2_ESM.zip › Questionnaire shake T1, T2 en-GBR1.docx]
