# Supplementary material for: PROMISE: effect of protein supplementation on fat-free mass preservation after bariatric surgery, a randomized double-blind placebo-controlled trial
Source: Trials. 2023 Nov 9;24:717. doi: 10.1186/s13063-023-07654-w (PMC10636856; doi:10.1186/s13063-023-07654-w)
Supplement: Supplementary file 3 — Additional file 3: Table 1a. Primary outcome: Percentage of fat free mass loss at 6 months post-surgery (intention-to-treat). Table 1b. Secondary outcome: Percentage of fat free mass loss at 6 months post-surgery (per protocol). Table 2a. Primary outcome: Percentage of fat free mass loss at 1, 3, (6) and 12 months post-surgery (intention-to-treat).§Table 2b. Secondary outcome: Percentage of fat free mass loss at 1, 3, (6) and 12 months post-surgery (per protocol).§ [file 13063_2023_7654_MOESM3_ESM.docx]

# **Supplement: Description of planned statistical analyses for the PROMISE outcome conform the ‘estimands’ framework**

**Table 1a: Primary outcome: Percentage of fat free mass loss at 6 months post-surgery (intention-to-treat).**

| **Objective:** To compare the effectiveness of a daily consumed clear protein powder shakes to placebo, given for a period of six months following bariatric surgery, on preserving fat free mass (FFM) during weight loss at 6 months after surgery in patients with morbid obesity undergoing a laparoscopic Roux-en-Y gastric bypass (LRYGB). | |
| --- | --- |
| **Estimand:** Percentage of FFM loss at 6 months: (FFM (kg) at 6 months – FFM (kg) at baseline) / (total weight (TW) (kg) at 6 months – TW (kg) at baseline) x 100%. | |
| **Treatment:** Arm A: Clear protein powder shake containing 20 grams of whey protein per serving daily dissolved in 200mL of water during the first six months after surgery.  Arm B: Clear placebo shake containing maltodextrine and being isocaloric with and having the same smell and taste as the protein shake, during the first six months after surgery. | |
| ESTIMAND | ANALYSIS |
| **Target population** | **Analysis set** |
| Patients meeting the IFSO criteria undergoing LRYGB. | All patients meeting eligibility criteria and being randomized. |
| **Variable** | **Outcome measure** |
| Percentage of fat free mass FFM loss (kg) at 6 months | (FFM (kg) at 6 months – FFM (kg) at baseline) / (total weight (kg) at 6 months – total weight (kg) at baseline) x 100%. FFM and TW will be assessed by multi-frequency bio-electrical impedance analysis (MF-BIA) using a Seca® MBCA 515. |
| **Handling of intercurrent events** | **Handling of missing data** |
| - Death / lost to follow-up (include in analysis if baseline measurements available, exclude otherwise.)  - Allocated treatment not applied (include in analysis according to the treatment arm randomized).  - Non-adherence to allocated treatment (include in analyses according to treatment arm randomized). | If missings in the primary outcome occur, these will be completed by imputation. The imputation model will include baseline covariates age, sex, physical activity, the intermediate outcome at 3 months, treatment group and all interactions with treatment group. |
| **Population-level summary measure** | **Analysis approach** |
| Mean difference in the percentage of FFM at 6 months between the two arms. | The analysis will be performed using the adaptive P-value combination test. The combination test will be used to combine the Z-statistics of the treatment effect obtained at both stages of the trial (interim analysis and final analysis) and ensures adequate control of the type-1 error rate under this adaptive design. The analysis will be conducted according to the intention-to-treat principle. |

**Table 1b: Secondary outcome: Percentage of fat free mass loss at 6 months post-surgery (per protocol).**

| **Objective:** To compare the effectiveness of a daily consumed clear protein powder shakes to placebo, given for a period of six months following bariatric surgery, on preserving fat free mass (FFM) during weight loss at 6 months after surgery in patients with morbid obesity undergoing a laparoscopic Roux-en-Y gastric bypass (LRYGB). | |
| --- | --- |
| **Estimand:** Percentage of FFM loss at 6 months: (FFM (kg) at 6 months – FFM (kg) at baseline) / (total weight (TW) (kg) at 6 months – TW (kg) at baseline) x 100%. | |
| **Treatment:** Arm A: Clear protein powder shake containing 20 grams of whey protein per serving daily dissolved in 200mL of water during the first six months after surgery.  Arm B: Clear placebo shake containing maltodextrine and being isocaloric with and having the same smell and taste as the protein shake, during the first six months after surgery. | |
| ESTIMAND | ANALYSIS |
| **Target population** | **Analysis set** |
| Patients meeting the IFSO criteria undergoing LRYGB. | All patients meeting eligibility criteria and being randomized. Cumulative intake of protein of at least 70% according to the self-reported calendar (treatment arm). In the placebo arm patients will be analyzed regardless of their intake. |
| **Variable** | **Outcome measure** |
| Percentage of fat free mass FFM loss (kg) at 6 months | (FFM (kg) at 6 months – FFM (kg) at baseline) / (total weight (kg) at 6 months – total weight (kg) at baseline) x 100%. FFM and TW will be assessed by multi-frequency bio-electrical impedance analysis (MF-BIA) using a Seca® MBCA 515. |
| **Handling of intercurrent events** | **Handling of missing data** |
| - Death / lost to follow-up (include in analysis if baseline measurements available, exclude otherwise.)  - Allocated treatment not applied (exclude from analysis if randomized to active arm).  - Non-adherence to allocated treatment (exclude from analysis if randomized to active arm and cumulative protein intake < 70%, include otherwise). | If missings in the primary outcome occur, these will be completed by imputation. The imputation model will include baseline covariates age, sex, physical activity, the intermediate outcome at 3 months, treatment group and all interactions with treatment group. |
| **Population-level summary measure** | **Analysis approach** |
| Mean difference in the percentage of FFM at 6 months between the two arms. | The analysis will be performed using the adaptive P-value combination test. The combination test will be used to combine the Z-statistics of the treatment effect obtained at both stages of the trial (interim analysis and final analysis) and ensures adequate control of the type-1 error rate under this adaptive design. The analysis will be conducted according to the per protocol principle. |

**Table 2a: Primary outcome: Percentage of fat free mass loss at 1, 3, (6) and 12 months post-surgery (intention-to-treat).^§^**

| **Objective:** To compare the effectiveness of a daily consumed clear protein powder shakes to placebo, given for a period of six months following bariatric surgery, on preserving fat free mass (FFM) during weight loss at 1, 3 (6) and 12 (x) months after surgery in patients with morbid obesity undergoing a laparoscopic Roux-en-Y gastric bypass (LRYGB). | |
| --- | --- |
| **Estimand:** Percentage of FFM loss at x months: (FFM (kg) at x months – FFM (kg) at baseline) / (total weight (TW) (kg) at x months – TW (kg) at baseline) x 100%. | |
| **Treatment:** Arm A: Clear protein powder shake containing 20 grams of whey protein per serving daily dissolved in 200mL of water during the first six months after surgery.  Arm B: Clear placebo shake containing maltodextrine and being isocaloric with and having the same smell and taste as the protein shake, during the first six months after surgery. | |
| ESTIMAND | ANALYSIS |
| **Target population** | **Analysis set** |
| Patients meeting the IFSO criteria undergoing LRYGB. | All patients meeting eligibility criteria and being randomized. |
| **Variable** | **Outcome measure** |
| Percentage of fat free mass FFM loss (kg) at x months | (FFM (kg) at x months – FFM (kg) at baseline) / (total weight (kg) at x months – total weight (kg) at baseline) x 100%. FFM and TW will be assessed by multi-frequency bio-electrical impedance analysis (MF-BIA) using a Seca® MBCA 515. |
| **Handling of intercurrent events** | **Handling of missing data** |
| - Death / lost to follow-up (include in analysis if baseline measurements available, exclude otherwise.)  - Allocated treatment not applied (include in analysis according to the treatment arm randomized).  - Non-adherence to allocated treatment (include in analyses according to treatment arm randomized). | Missings in the outcome vector will be automatically accounted for by the longitudinal model, which allows subjects to remain in the analysis, as long as the outcome has been measured at least onat one occasion. Unbiased estimates can still be obtained under the missing at random (MAR) assumption. |
| **Population-level summary measure** | **Analysis approach** |
| Mean difference in the percentage of FFM at x months between the two arms. | The analysis will be performed by using a generalized least squares model (a type of repeated measures model for continuous outcomes). An unstructured correlation structure for the repeated measurements over time within each subject will be assumed. The treatment effect for the follow-up visits will be modelled by including covariates for treatment, visit and its interactions. The analysis will be conducted according to the intention-to-treat principle. |

**^§^** Outcomes total weight loss, fat mass loss, BMI and hand grip strength will be analyzed in a similar fashion.

**Table 2b: Secondary outcome: Percentage of fat free mass loss at 1, 3, (6) and 12 months post-surgery (per protocol).^§^**

| **Objective:** To compare the effectiveness of a daily consumed clear protein powder shakes to placebo, given for a period of six months following bariatric surgery, on preserving fat free mass (FFM) during weight loss at 1, 3 (6) and 12 (x) months after surgery in patients with morbid obesity undergoing a laparoscopic Roux-en-Y gastric bypass (LRYGB). | |
| --- | --- |
| **Estimand:** Percentage of FFM loss at 6 months: (FFM (kg) at 6 months – FFM (kg) at baseline) / (total weight (TW) (kg) at 6 months – TW (kg) at baseline) x 100%. | |
| **Treatment:** Arm A: Clear protein powder shake containing 20 grams of whey protein per serving daily dissolved in 200mL of water during the first six months after surgery.  Arm B: Clear placebo shake containing maltodextrine and being isocaloric with and having the same smell and taste as the protein shake, during the first six months after surgery. | |
| ESTIMAND | ANALYSIS |
| **Target population** | **Analysis set** |
| Patients meeting the IFSO criteria undergoing LRYGB. | All patients meeting eligibility criteria and being randomized. Cumulative intake of protein of at least 70% according to the self-reported calendar (treatment arm). In the placebo arm patients will be analyzed regardless of their intake. |
| **Variable** | **Outcome measure** |
| Percentage of fat free mass FFM loss (kg) at x months | (FFM (kg) at x months – FFM (kg) at baseline) / (total weight (kg) at x months – total weight (kg) at baseline) x 100%. FFM and TW will be assessed by multi-frequency bio-electrical impedance analysis (MF-BIA) using a Seca® MBCA 515. |
| **Handling of intercurrent events** | **Handling of missing data** |
| - Death / lost to follow-up (include in analysis if baseline measurements available, exclude otherwise.)  - Allocated treatment not applied (exclude from analysis if randomized to active arm).  - Non-adherence to allocated treatment (exclude from analysis if randomized to active arm and cumulative protein intake < 70%, include otherwise). | Missings in the outcome vector will be automatically accounted for by the longitudinal model, which allows subjects to remain in the analysis, as long as the outcome has been measured at least onat one occasion. Unbiased estimates can still be obtained under the missing at random (MAR) assumption. |
| **Population-level summary measure** | **Analysis approach** |
| Mean difference in the percentage of FFM at x months between the two arms. | The analysis will be performed by using a generalized least squares model (a type of repeated measures model for continuous outcomes). An unstructured correlation structure for the repeated measurements over time within each subject will be assumed. The treatment effect for the follow-up visits will be modelled by including covariates for treatment, visit and its interactions. The analysis will be conducted according to the per protocol principle. |

**^§^** Outcomes total weight loss, fat mass loss, BMI and hand grip strength will be analyzed in a similar fashion.
